# Correction: Preparation and properties of a novel alginate/carrageenan crosslinked coordination polymer and evaluation of the antibacterial, antioxidant and anticancer potential of its Co(ii), and Cr(iii) polymeric complexes

**DOI:** 10.1039/d5ra90045j

**Published:** 2025-04-17

**Authors:** Maged S. Al-Fakeh, Nora S. Al-Subaie, Yassine EL-Ghoul, Zeineb Hamden

**Affiliations:** a Department of Chemistry, College of Science, Qassim University Buraidah 51452 Saudi Arabia; b Taiz University Taiz 3086 Yemen; c Department of Chemistry, College of Science, University of Bisha Bisha 61922 Saudi Arabia; d Textile Engineering Laboratory, University of Monastir Monastir 5019 Tunisia; e Laboratory of Interfaces and Advanced Materials, Faculty of Sciences of Monastir, University of Monastir Monastir 5000 Tunisia

## Abstract

Correction for ‘Preparation and properties of a novel alginate/carrageenan crosslinked coordination polymer and evaluation of the antibacterial, antioxidant and anticancer potential of its Co(ii), and Cr(iii) polymeric complexes’ by Maged S. Al-Fakeh *et al.*, *RSC Adv.*, 2024, **14**, 38934–38943, https://doi.org/10.1039/D4RA06818A

The authors regret the omission of two affiliations (affiliation *b* and *d* shown here) from the original manuscript. The corrected list of affiliations is as shown here.

The Royal Society of Chemistry apologises for these errors and any consequent inconvenience to authors and readers.

